# Sudden onset of severe pulmonary edema after emergency cesarean section

**DOI:** 10.1186/s40981-016-0049-2

**Published:** 2016-09-22

**Authors:** Yuichiro Shimoyama, Toshiyuki Sawai, Osamu Umegaki, Tomoyuki Agui, Noriko Kadono, Junko Nakahira, Toshiaki Minami

**Affiliations:** 1 Department of Anesthesiology, Osaka Medical College, 2-7 Daigaku-machi, Takatsuki, Osaka 569-8686 Japan; 2 Department of Surgery, Osaka Medical College, Takatsuki, Japan

## Abstract

Mitral valve stenosis (MS) associated with rheumatic disease no longer represents a major heart problem during the perinatal period in Japan. Here we present a case of acute heart failure due to MS after emergency cesarean section (CS). The patient was transferred due to the development of fetal distress at 36 weeks gestation and underwent an emergency CS under general anesthesia. She developed acute heart failure immediately postoperatively and was diagnosed with MS associated with pulmonary artery hypertension for the first time. She underwent percutaneous transvenous mitral commissurotomy and was discharged from our hospital in good condition.

## Findings

**Fig. 1:**
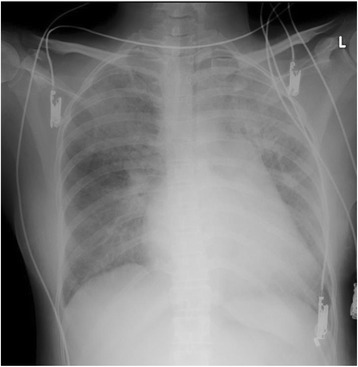
Chest X-ray showing the butterfly pattern of pulmonary edema

**Fig. 2:**
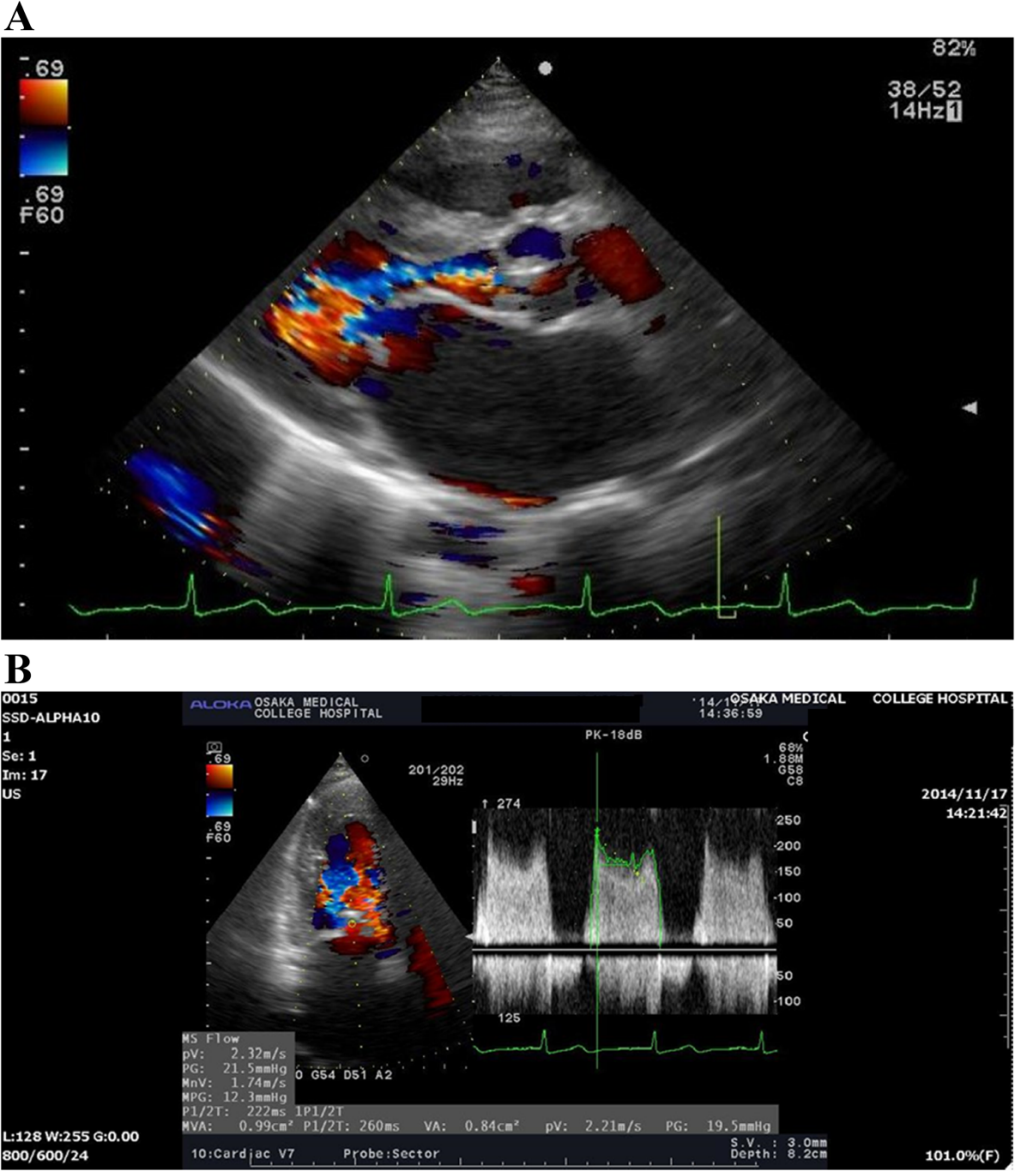
**a** Parasternal long-axis view with color Doppler assessment showing severe mitral valve stenosis. **b** Mitral valve area was 0.99 cm^2^ as measured by the pressure half-time method

A 37-year-old patient (150 cm, 52 kg) originally from the Philippines with no remarkable history was transferred to our hospital at 36 weeks and 3 days gestation after having diagnosed with abruptio placentae. Upon admission, she underwent an emergency cesarean section (CS) under general anesthesia due to the development of fetal distress. The patient was induced with 100 mg IV propofol, 100 μg fentanyl, and 50 mg rocuronium and then underwent tracheal intubation. Anesthesia (sevoflurane 1 % and remifentanil 3 μg/kg/min) was maintained. Her hemodynamics and respiratory condition were both within normal ranges throughout the intraoperative period. She delivered a female live-born baby, with 1- and 5-min APGAR scores of 1 and 6, respectively, and a birth weight of 2626 g. The time from hospital arrival to delivery was 13 min. The baby recovered stably in the neonatal ICU. The patient was extubated and transferred to the ICU; her central venous pressure was 3 mmHg, heart rate 122 beats/min, arterial blood pressure 127/62 mmHg, and oxygen saturation 100 % (8 L facemask). The patient was administered a total of 1400 mL of fluid (700 mL acetate Ringer’s solution, 700 mL hydroxyethyl starch) during the operation. Her postoperative chest X-ray was unremarkable. After 15 min (102 min after delivery), however, the patient complained of dyspnea and her oxygen saturation gradually decreased to 93 %. Despite oxygen therapy, oxygen saturation further decreased to 67 % (15 L reservoir mask) rapidly. We immediately re-intubated the patient to manage respiratory failure; frothy, blood-stained sputum was aspirated through the intubation tube. Physical examination revealed a diastolic murmur at the mitral focus and bilateral pulmonary rales. An ECG showed a sinus tachycardia (HR145 beats/min), and the radiograph of the chest showed the butterfly pattern of pulmonary edema (Fig. 1). Although only minor bleeding through the drains was noted, her blood pressure and circulation were unstable (shock index >1; HR85 beats/min, BP53/34 mmHg). After administration of dopamine (3–5 μg/kg/min), hemodynamics showed an improvement. At this time, the patient was started on intravenous furosemide (20–40 mg/day) and 100 mg/day potassium canrenoate for the treatment of heart failure. After her general condition stabilized, we performed transthoracic echocardiography (TTE). TTE showed mitral valve stenosis (MS) with mild mitral valve regurgitation (Fig. 2a), with a mitral valve area (MVA) of 0.99 cm^2^ as measured by the pressure half-time method (Fig. 2b). Right ventricular systolic pressure (RVSP) was estimated to be 118 mmHg (CVP 15 mmHg) from the tricuspid valve regurgitation velocity (5 m/s). The final diagnosis was decompensated heart failure caused by post-rheumatic MS after delivery. After diuretic therapy, her pulmonary edema due to acute cardiac failure resolved. She was extubated on the following day and eventually underwent percutaneous transvenous mitral commissurotomy (PTMC), due to the Wilkins score, which was lower than 8. After the second inflation of the Inoue balloon, MVA increased to 1.34 cm^2^ and RVSP decreased by 50 %. The patient was discharged a few days after PTMC in good condition.

PTMC using the Inoue balloon catheter has become an accepted treatment option for patients with severe symptomatic MS. PTMC provides palliation for pregnant women with MS, with a reported success rate of nearly 100 %. Successful balloon valvuloplasty increases the valve area to >1.5 cm^2^ without a substantial increase in mitral regurgitation [1]. A report showed that mean MVA before the procedure (range, 0.75 to 1.2 cm^2^) increased after the procedure to 1.7 to 2.2 cm^2^. These results are comparable to the results reported on non-pregnant patients with MS [2]. In the present case, MS was not diagnosed before the patient was transferred to our hospital, and the patient presented with no pathological events, such as congestive heart failure and arrhythmia. Moreover, there was no time to diagnose MS from the time of arrival to CS due to fetal distress. Consequently, appropriate medication or invasive MS intervention was not performed until the postpartum period.

In the present case, the patient was diagnosed with MS associated with pulmonary edema for the first time during the postpartum period. It can explain decompensation in a postpartum woman with critical MS as follows. The sudden increase in the pre-load immediately following delivery, due to autotransfusion from the uterus, may flood the central circulation, resulting in severe pulmonary edema. In addition, given that autotransfusion of blood continues for 24–72 h post-delivery, there is an extended risk of pulmonary edema for several days post-delivery [3]. Maternal mortality occurs most frequently during this time period [4]. However, MS onset was within 2 h after delivery. It might be too early to explain only with this theory. In the present case, we performed general anesthesia in the patient as she underwent rapid induction. General anesthesia is disadvantageous as it increases pulmonary arterial pressure and tachycardia during endotracheal intubation. In addition, the harmful effects of positive pressure ventilation on venous return may ultimately lead to heart failure [5]. Regional anesthesia has proven to be a safe technique in cardiac patients undergoing CS [3]. That said, we have no information regarding MS development with anesthesia induction, so it may be prudent to perform regional anesthesia for now.

We experienced and managed a patient who was diagnosed with MS associated with pulmonary edema for the first time after emergency CS. An adequate perioperative management by a multidisciplinary team comprising an anesthesiologist, cardiologist, critical care physician, and obstetrician may lead to a reduction in perinatal mortality and morbidity.

## Abbreviations

MS: Mitral valve stenosis
CS: Cesarean section
PTMC: Percutaneous transvenous mitral commissurotomy
TTE: Transthoracic echocardiography
RVSP: right ventricular systolic pressure
